# Beyond the hype: a psychological perspective on AI chatbots

**DOI:** 10.3389/fpsyg.2026.1766427

**Published:** 2026-03-05

**Authors:** Xuanxuan Zhou, Chengli Li

**Affiliations:** 1 Wenzhou Business College, Wenzhou, China; 2 Sichuan University Jinjiang College, Meishan, China

**Keywords:** a psychological perspective, affective lens, AI chatbots, cognitive lens, language education, social lens

## 1 Introduction

The accelerating evolution of artificial intelligence (AI) has reshaped the educational landscape, driving the adoption of intelligent tools across education environments (Msambwa et al., 2025; Walter, 2024). Within this broader technological shift, AI chatbots have gained particular prominence in language education. Empirical studies consistently highlight their benefits, demonstrating improved vocabulary development, heightened engagement, and enhanced instructional efficiency (Almasri, 2024; Zhang and Huang, 2024; O'Neill et al., 2025). Their capacity to deliver interactive, personalized, and on-demand communicative practice offers learners and teachers opportunities to engage in meaningful language use beyond the constraints of traditional classrooms (Pan et al., 2024; Li et al., 2025). This interactive engagement is consistent with human–machine communication (HMC) (Guzman and Lewis, 2020; Guzman et al., 2023), as learners tend to attribute social presence and communicative intent to chatbots, perceiving them as meaningful interlocutors.

Yet the expanding presence of AI chatbots has generated debate, primarily due to their complex psychological implications for users. Evidence shows that Over-reliance on AI can undermine students' cognitive skills, critical thinking, creativity, and motivation, while simultaneously intensifying teachers' responsibilities and pressures associated with human–AI collaboration (Kim, 2024; Zhai et al., 2024). Other studies caution that substituting human interaction with AI chatbot communication may erode social support, weaken sense of belonging, and ultimately undermine wellbeing, academic persistence, and overall success (Crawford et al., 2024; Klimova and Pikhart, 2025). In contrast, an emerging body of research identifies clear psychological advantages: interactions with chatbots have been associated with reduced anxiety, increased confidence, and strengthened motivation in language learning (Wei et al., 2025; Zhang, 2026).

These contrasting findings underscore a crucial point: the impact of AI chatbots in language education cannot be fully understood through a purely technological lens. Despite growing evidence on the psychological effects of AI chatbots, existing findings remain fragmented, underscoring the need for a more critical, psychology-driven reconsideration of their role in language education. To move beyond the prevailing hype and surface-level enthusiasm, it is essential to analytically deconstruct the role of AI chatbots in language education through the lens of psychology. To be more specific, to study AI chatbots through a psychological lens in digital environments, this study examines them from three interrelated perspectives: cognitive, affective, and social lens (Mayer, 2024; Schneider et al., 2022). Unlike existing studies that primarily evaluate the effectiveness or feasibility of AI chatbots, this opinion argues for a psychological reorientation of how chatbots are conceptualized in language education. Specifically, it reframes AI chatbots as interactional presences that shape cognitive processing, emotional regulation, and social engagement in interconnected ways.

## 2 AI chatbots in language education through three psychological lenses

### 2.1 Cognitive psychological lens: enhanced attention but risks of cognitive overload

From a cognitive psychological perspective, AI chatbots appear to support learners' attentional focus and knowledge processing. Studies have shown that chatbot-assisted interactions can increase learners' engagement, deepen topic exploration, and scaffold comprehension in academic tasks such as research proposal writing (Smirnova, 2025). Similarly, voice-based chatbots have been found to sustain learners' attention and facilitate meaningful practice, partly due to their immediate, adaptive responses (Koç and Savaş, 2025).

However, these reported benefits often center on surface-level behavioral indicators—such as time-on-task or perceived engagement—rather than examining whether chatbots genuinely improve cognitive processing quality. Psychological risks are also evident. Chatbot-generated inaccuracies may create confusion, increase extraneous cognitive load, and weaken autonomous problem-solving (Huete-García and Tarp, 2024). The tendency of large language models to generate factually unreliable yet coherent responses further exacerbates this cognitive burden (Lappin, 2024). Moreover, learners may become behaviorally dependent on chatbots for tasks requiring generative thinking, potentially reducing opportunities for deeper cognitive engagement (Feng et al., 2025). Taken together, while chatbots may enhance cognitive engagement, their impact on cognitive quality remains unclear, warranting more critical scrutiny.

### 2.2 Affective psychological lens: reduced anxiety but uncertain long-term outcomes

From an affective psychological lens, studies have shown that chatbots reduce learner anxiety and foster low-stress learning environments. Studies report reductions in foreign language anxiety, increases in confidence, and greater willingness to communicate when learners interact with AI chatbots rather than human partners (Kohnke et al., 2023; Zhang, 2026; Kim and Su, 2024). Furthermore, AI chatbots validate learners' non-native language output—accurately interpreting diverse accents and learner varieties—helping reduce reliance on native-speaker norms and boosting perceived competence (Lee et al., 2025). Yet, affective benefits still require careful interpretation. As many studies rely heavily on self-reported perceptions, their findings risk conflating positive emotional reactions with genuine learning improvement. Reduced anxiety may also lead learners to prefer chatbot-based communication and avoid real social interaction, limiting their ability to cope with authentic communicative pressure. Moreover, the short-term motivational boosts reported in the literature offer limited insight into whether chatbots sustain long-term engagement or language development. Thus, while chatbots often enhance emotional comfort, the psychological implications for long-term application remain insufficiently explored.

### 2.3 Social psychological lens: increased social connectedness but erosion of human interaction

From a social psychological perspective, AI chatbots can shape learners' sense of connection, belonging, and relational trust. Politeness-enabled chatbots, for instance, have been shown to increase users' trust, reduce defensiveness, and foster a sense of social connectedness, contributing to more positive interactional experiences (Brummernhenrich et al., 2025). These socially meaningful interactions also reveal a dynamic socio-technical relationship, suggesting that attention to visual and emotional design elements can enhance engagement and overall user experience (Haqqu et al., 2025). Learners' perceptions of chatbots have also been linked to improvements in self-perceived competence and writing engagement (Mills et al., 2025). In these cases, chatbots serve as supportive, non-judgmental social partners.

However, social psychological research also warns of potential relational costs. When chatbots replace human-human interaction, they may inadvertently reduce social support, weaken learners' sense of belonging, and ultimately undermine wellbeing and academic persistence (Crawford et al., 2024; Klimova and Pikhart, 2025). Moreover, reliance on AI-mediated interaction risks diminishing opportunities for genuine social negotiation—an essential component of language learning. In broader terms, ethical concerns related to privacy, data misuse, and algorithmic bias can further erode trust in AI systems (Zhang and Yu, 2025; Labadze et al., 2023; Yigci et al., 2025). These studies suggest that while AI chatbots can foster social connectedness in controlled contexts, their long-term social implications in educational settings remain ambiguous and require careful consideration.

In this way, the cognitive, affective, and social dimensions discussed above should not be understood as independent effects of AI chatbot use. Specifically, reduced anxiety may lower cognitive load and encourage engagement, while perceived social presence can enhance motivation and sustained participation. At the same time, cognitive reliance on chatbots may gradually reshape learners' emotional comfort and preferences for social interaction. Overall, these dynamics suggest that the psychological impact of AI chatbots in language education emerges from the interplay of cognitive, affective, and social processes rather than from isolated mechanisms.

## 3 Future directions

Moving forward, current studies on AI chatbots in language education have generated valuable insights into cognitive engagement, emotional responses, and social experiences (Ebadi and Amini, 2024; Zou et al., 2025). To advance understanding in this area, several directions warrant further exploration.

First of all, although this study provides insights into users' cognitive, emotional, and social experiences, there remains a need to better understand their interrelations in chatbot-mediated teaching. In the future, studies can investigate how different types of chatbot interactions influence cognitive load (Sweller, 1988), emotional engagement (Fredricks et al., 2004), and social connectedness (Gunawardena, 1995). Future studies could employ experimental, longitudinal, or mixed-method designs to examine how different types of chatbot interactions influence learners' cognitive load, emotional engagement, motivation, social connectedness, and trust across diverse educational contexts, such as university language courses, online learning, and blended learning environments. Such studies would provide a deeper understanding of the psychological pathways through which chatbots affect language education.

Second, pedagogical practice requires designs that intentionally scaffold these three domains. Rather than allowing chatbots to replace higher-order thinking, instruction should encourage students and teachers to interrogate, compare, and refine chatbot outputs. Affective scaffolding should balance anxiety-reducing practice with authentic communicative demands (Krashen, 1982) to prevent overreliance on low-pressure environments. Socially, chatbots should be positioned as supplementary partners within a community of inquiry (Garrison et al., 1999) rather than substitutes for peers or teachers. In particular, chatbots can be integrated as preparatory or reflective tools to support idea generation, low-stakes rehearsal, and the interpretation of feedback. Core processes of meaning-making, negotiation, and evaluative judgment should remain anchored in instructional interaction. Future research can also evaluate how different instructional designs, such as preparatory or reflective chatbot activities, affect students' engagement, anxiety reduction, and reflective use, using classroom observation, interaction analysis, and learning outcome assessment. Thus, instructional integration foregrounds human agency and interaction, with AI chatbots functioning as structured support alongside essential pedagogical relationships.

Finally, institutional policies should account for the psychological implications of AI-assisted education. Clear usage guidelines, professional training, and support systems can help teachers manage the cognitive, emotional and psychological demands of supervising AI-mediated activities. Ethical safeguards on data privacy, informed consent, and bias detection are also necessary to uphold users' wellbeing and trust. Teachers play a critical role in guiding students' engagement with AI chatbots by monitoring cognitive reliance, facilitating emotional regulation, and fostering reflective use. Educational institutions are responsible for establishing governance structures, providing professional training, and setting ethical guidelines that determine how and why chatbots are implemented in formal learning contexts.

## 4 Conclusion

This research argues that much of the current enthusiasm surrounding AI chatbots in language education risks oversimplifying their pedagogical value. By moving beyond the prevailing hype and reframing chatbots through cognitive, affective, and social psychological lenses, this article shows that their impact is far more nuanced than commonly claimed. While evidence demonstrates clear benefits—such as enhanced attentional focus, reduced anxiety, and increased social connectedness—equally important psychological risks emerge, including cognitive overload, avoidance of authentic communication, and weakened human interaction. The contribution of this study lies in synthesizing these fragmented findings into a coherent psychological interpretation, offering a more balanced and comprehensive understanding of how chatbots actually shape language education. This perspective underscores the need for future research to examine underlying psychological processes rather than surface-level perceptions, encourages pedagogical designs that intentionally scaffold cognitive, emotional, and social development, and calls for institutional policies that ensure ethical, transparent, and human-centered AI use. Crucially, the ethical use of AI chatbots in language education requires institutional governance regarding data ownership, transparency in system decision-making, and mechanisms to safeguard user trust. Without such structures, potential psychological benefits may be undermined by concerns over privacy, accountability, and power asymmetries. Taken together, a psychological reorientation enables the field to evaluate AI chatbots not as inherently beneficial or harmful tools, but as complex cognitive-affective-social technology whose value depends on how they are designed, integrated, and governed.
